# Ligand-Mediated Endocytosis and Trafficking of the Insulin-Like Growth Factor Receptor I and Insulin Receptor Modulate Receptor Function

**DOI:** 10.3389/fendo.2014.00220

**Published:** 2014-12-17

**Authors:** Alaide Morcavallo, Manuela Stefanello, Renato V. Iozzo, Antonino Belfiore, Andrea Morrione

**Affiliations:** ^1^Departments of Urology, Sydney Kimmel Cancer Center, Thomas Jefferson University, Philadelphia, PA, USA; ^2^Department of Health Sciences and Endocrinology, University Magna Graecia of Catanzaro, Catanzaro, Italy; ^3^Department of Pathology, Anatomy and Cell Biology, Sydney Kimmel Cancer Center, Thomas Jefferson University, Philadelphia, PA, USA; ^4^Cancer Cell Biology and Signaling Program, Sydney Kimmel Cancer Center, Thomas Jefferson University, Philadelphia, PA, USA; ^5^Biology of Prostate Cancer Program, Sydney Kimmel Cancer Center, Thomas Jefferson University, Philadelphia, PA, USA

**Keywords:** IGF-IR, IR, endocytosis, trafficking, signaling

## Abstract

The insulin-like growth factor system and its two major receptors, the IGF receptor I (IGF-IR) and IR, plays a central role in a variety of physiological cellular processes including growth, differentiation, motility, and glucose homeostasis. The IGF-IR is also essential for tumorigenesis through its capacity to protect cancer cells from apoptosis. The IR is expressed in two isoforms: the IR isoform A (IR-A) and isoform B (IR-B). While the role of the IR-B in the regulation of metabolic effects has been known for several years, more recent evidence suggests that the IR, and in particular the IR-A, may be involved in the pathogenesis of cancer. Ligand-mediated endocytosis of tyrosine-kinases receptors plays a critical role in modulating the duration and intensity of receptors action but while the signaling pathways induced by the IGF-IR and IR are quite characterized, very little is still known about the mechanisms and proteins that regulate ligand-induced IGF-IR and IR endocytosis and trafficking. In addition, how these processes affect receptor downstream signaling has not been fully characterized. Here, we discuss the current understanding of the mechanisms and proteins regulating IGF-IR and IR endocytosis and sorting and their implications in modulating ligand-induced biological responses.

## Introduction

**Figure 1:**
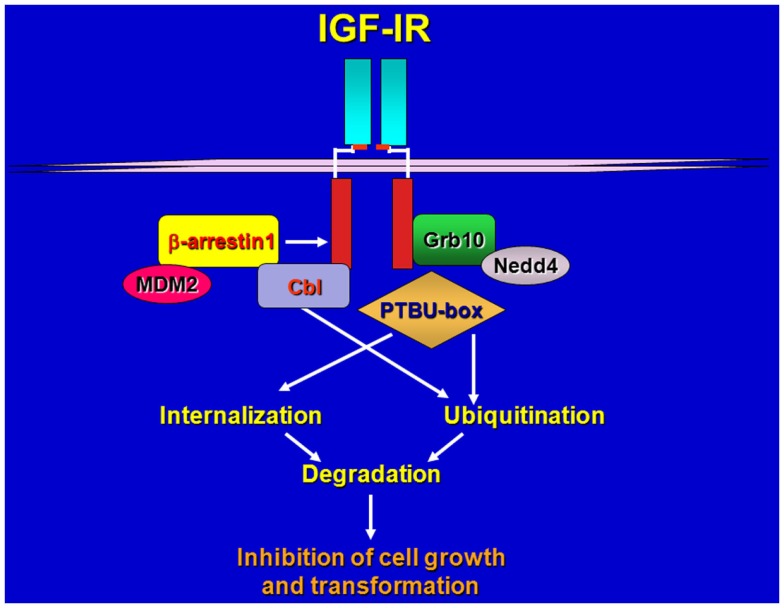
**Schematic draws of IGF-IR regulation by various ligases and adaptors**. Upon ligand-stimulation ubiquitin ligases complex with the IGF-IR either directly or through adaptor proteins, promoting receptor ubiquitination, internalization, and sorting for degradation.

The IGF receptor I (IGF-IR) and its cognate ligands insulin-like growth factors I and II (IGF-I and IGF-II) play an essential role in modulating mammalian growth *in vitro* (1, 2) and *in vivo* (3–5). The IGF-IR, IGF-I, and IGF-II are often deregulated in cancer and may have a critical function not only in the early phases of tumor initiation but also in cancer progression and resistance to therapies (6–9). IGF-II, and to a lesser extent IGF-I, binds to the isoform A of the insulin receptor (IR-A), which has high homology to the IGF-IR (10, 11) (Figure 1). The IR-A is the fetal form of the IR and mediates primarily mitogenesis upon IGF-II or insulin activation (11–13) and is also implicated in transformation (14, 15), while the second IR isoform (IR-B) is involved in glucose homeostasis of insulin-sensitive organs (11, 14). Prevalent expression of the IR-A over the IR-B has been discovered in several cancer models, and an autocrine proliferative loop between IGF-II and the IR-A has been detected in malignant thyrocytes, breast cancer, and sarcoma cells (16–19).

Ligand-dependent endocytosis and sorting for degradation of receptor-tyrosine kinases (RTKs) has recently emerged as a critical step in modulating the duration and intensity of receptor biological activities (20, 21). Ligand-mediated polyubiquitination of RTKs targets them for degradation to the lysosomal pathway, to mediate receptor down-regulation (20). Recent reports have suggested that the EGF-R and the PDGFR may not be polyubiquitinated but rather monoubiquitinated at multiple sites (multiubiquitination), and this modification is sufficient to ensure receptor sorting and degradation (22, 23).

While the mechanisms regulating EGF-R and PDGFR endocytosis have been extensively studied, very little is still understood about endocytosis of the IGF-IR and IR. In this review, we will summarize recent advances in understanding the mechanisms regulating IGF-IR and IR-A ubiquitination, endocytosis, and sorting, and discuss the role that different cognate ligands play in regulating these processes.

## IGF-IR Ubiquitination, Endocytosis, and Trafficking

Our and other laboratories identified the adaptor protein Grb10 as a novel IGF-IR and IR binding partner (24, 25) and established an important role for this adapter in the regulation of IGF-IR-dependent cell proliferation (26). We later discovered that Grb10 constitutively associates with the Hect E3 ubiquitin ligase Nedd4 (27) and promotes IGF-I-dependent multiubiquitination of the IGF-IR (28, 29), internalization through clathrin-dependent and -independent pathways (29) and subsequent degradation of the IGF-IR through a mechanism sensitive to inhibitors of both the proteosomal and lysosomal pathways (28, 29). IGF-IR down-regulation has been associated with the ubiquitin–proteasome pathway in lung cancer cells (30) while Nedd4-mediated and LDL-induced IGF-IR ubiquitination and degradation of the IGF-IR likely occurs through a proteosome-independent pathway (31).

Our work provided the first evidence of the involvement of a Hect E3 ligase in promoting ubiquitination of a RTK, and confirmed the critical role that receptor endocytosis plays in regulating IGF-IR downstream signaling (32) and biological responses (26). However, additional ubiquitin ligases have been shown to regulate ligand-induced ubiquitination of the IGF-IR in different cellular systems, utilizing Grb10-independent mechanisms.

Girnita et al. (33) discovered that the ubiquitin ligase Mdm2 promotes ubiquitination of the IGF-IR (33) via the adaptor function of β-arrestin1 protein (34). Mdm2 is a ring-finger ubiquitin ligase, which also regulates p53 ubiquitination and stability (35, 36), therefore, these data suggest that the role of Mdm2 in promoting ubiquitination of the IGF-IR is likely more relevant in cellular backgrounds where decreased levels of p53 may enhance Mdm2 availability and action outside the nucleus.

The ring-finger E3 cCbl has been also identified as a novel IGF-IR ubiquitin ligase but it has distinct role from Mdm2 in receptor ubiquitination and endocytosis (37). Upon ligand-stimulation, both Mdm2 and cCbl are recruited to the IGF-IR but while Mdm2 promoted polyubiquitination through Lys 63 linkages, cCbl-mediated polyubiquitination occurred through Lys-48 chains. In addition, c-Cbl-mediated IGF-IR ubiquitination was only detectable after cell stimulation with high concentrations of IGF-I (50–100 ng/ml) whereas Mdm2-induced ubiquitination was detectable at physiological concentrations of ligand (37). Importantly, while Mdm2 promoted internalization of the ubiquitinated-IGF-IR through clathrin-dependent endocytosis, cCbl induced receptor internalization via a caveolar route (37). While it has been clearly established that ligand-induced Mdm-2-mediated ubiquitination of the IGF-IR targets the receptor for proteosomal degradation (33, 38), whether Cbl-mediated ubiquitination of the IGF-IR modulates receptor sorting for degradation or recycling has not been clearly defined.

More recent data have shown that an engineered ubiquitin ligase PTBU-box can promote the ubiquitination and degradation of IGF-IR and IR, and thus effectively inhibit *in vitro* and *in vivo* tumorigenesis of liver cancer HepG2 and cervical cancer HeLa cells that over-express IGF-IR and IR (39).

Because different ubiquitin ligase proteins have been implicated in mediating the ubiquitination of the IGF-IR (28, 33, 37, 39) we can speculate that different complexes may have different abilities in promoting either polyubiquitination or multiubiquitination of the receptor depending on either cell background or tumor model thus differentially affecting receptor sorting, stability, and biological activity (Figure 1).

The effects of ligand-mediated IGF-IR internalization on receptor signaling are quite complex. While IGF-IR endocytosis and subsequent receptor degradation negatively regulates downstream biological responses (26, 29), early events of IGF-IR internalization might play instead an important role in modulating receptor signaling. Indeed, IGF-IR internalization was required for Shc activation but not for IRS-1 phosphorylation, which was mediated by both cell surface and endosomal IGF-IR (32). Inhibition of clathrin and caveolin-dependent endocytosis impairs IGF-IR signaling in Ewing’s sarcoma cells, while caveolin-1 down-regulation inhibits IGF-IR internalization and receptor signal transduction in H9C2 rat cardiomyoblasts and HaCat cells (40–42). In addition, MDM2 and β-arrestin1 modulates IGF-IR-dependent ERK activation (38).

While IGF-I-induced IGF-IR activation is a critical step for receptor ubiquitination and endocytosis, there is also evidence of ligand-independent IGF-IR ubiquitination mediated by anti-IGF-IR neutralizing antibodies, which promote very efficient internalization and target the receptor for degradation (43). H10H5 antibody induced robust IGF-IR ubiquitination, which consisted of polyubiquitin chains (Lys-48 and Lys-29) mapped to tyrosine 1138 and tyrosine 1141 in the activation loop of the IGF-IR (43). The same tyrosine residues are ubiquitinated after IGF-I stimulation albeit to a lesser extent (43). Significantly, the identification of a breast cancer cell line defective in IGF-IR ubiquitination suggested a possible tumor resistance mechanism to overcome targeted IGF-IR down-regulation in cancer (43).

## IR Ligands Differentially Modulate Receptor Ubiquitination, Endocytosis, and Trafficking

Early studies established that the IR, similarly to other RTKs, is internalized from the cell surface upon ligand stimulation (44). The majority of work has been focused on endocytosis of the IR through clathrin-dependent pathways (44, 45), but other reports have also pointed out that additional pathways may contribute in modulating IR internalization (46). More recent data have in fact demonstrated a role of caveolae in mediating rapid insulin-dependent internalization of the IR (47, 48). The majority of these experiments was performed in adipocytes, which preferentially express the IR-B isoform (11, 14) and exclusively upon insulin stimulation.

The mechanisms and proteins regulating IR endocytosis are still poorly understood. The nine putative transmembrane protein LMBD1, encoded by the limb region 1 (LMBR1) domain containing one gene (*lmbrd1*), has been show to play an important role in regulating the endocytosis of the IR (49). LMBD1 co-internalized with the IR in clathrin-containing vesicles and LMBD1 depletion attenuated IR endocytosis, resulting in the perturbation of the IR recycling pathway and consequential enhancement of the IR signaling cascade (49).

Using mouse embryonic fibroblasts (MEFs) derived from wild type (WT) and PKCε-deficient [PKCε(−/−)] mice Pedersen et al. (50) demonstrated that PKCε modulated IR localization and trafficking by regulating CEACAM1 (50), a receptor substrate previously shown to modulate insulin clearance (51).

Recent work has pointed out a central role of the muscle-specific E3 ubiquitin ligase mitsugumin 53 (MG53; also called TRIM72) in promoting ubiquitin-dependent IR and IRS-1 degradation, which is associated with reduced IR signaling, insulin resistance, and metabolic disorders (52).

Our laboratories have recently focused on the mechanisms of action of the IR-A and the ability of different IR-A ligands to regulate IR-A-dependent signaling and mitogenesis.

**Figure 2:**
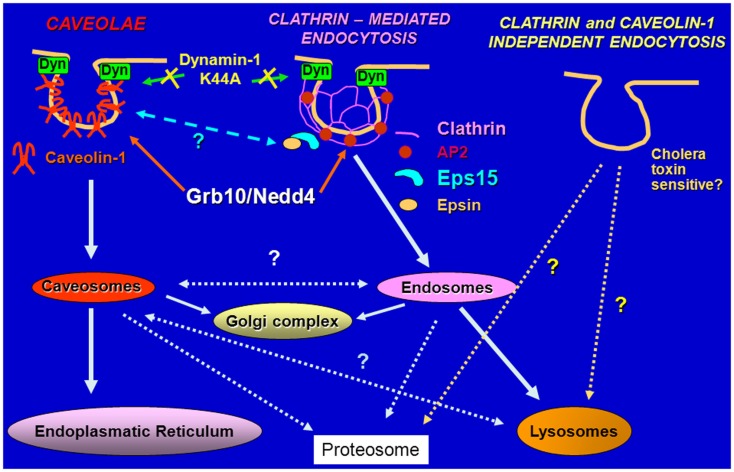
**Schematic diagram of pathways regulating endocytosis**. The IGF-IR and the IR are internalized in ligand-dependent manner through both clathrin-dependent and -independent pathways, sorted into early endosomes and either targeted for degradation in the lysosomes or recycled to the cell surface.

Because IGF-II is mitogenic through the IR-A at a comparable rate if not even higher than insulin, in spite of an affinity for the IR-A 3–5-fold lower than insulin and a reduced ability to promote receptor phosphorylation and activation of downstream effectors (11, 12), we undertook studies to test the hypothesis that insulin and IGF-II could affect biological responses by differentially regulating IR-A endocytosis and trafficking. Taking advantage of the unique model of R^−^/IR-A cells, which lack the IGF-IR (53) and were engineered to express solely the IR-A (54), we demonstrated that insulin and IGF-II considerably differ in their ability to regulate IR-A and downstream effectors trafficking and stability (55). Indeed, insulin stimulation of R^−^/IR-A cells promoted IR-A internalization, which was instead only modestly affected by IGF-II stimulation. Significantly, the difference in internalization was not due to IR-A ubiquitination, which was comparable in IGF-II and insulin-stimulated R^−^/IR-A cells (55). As control, we used the insulin analog NMeTyr^B26^-insulin, which has lower affinity than insulin for IR-A, and demonstrated that it promoted IR-A phosphorylation, internalization, and proliferation at a rate comparable to IGF-II. More importantly, we discovered that prolonged stimulation of R^−^/IR-A cells with insulin, but not with IGF-II or NMeTyr^B26^-insulin, targeted the IR-A and IRS-1 for degradation (55). We also elucidated the pathways of IR-A endocytosis and sorting and showed that upon insulin or IGF-II stimulation, the IR-A was internalized through clathrin-dependent and independent pathways, but only the clathrin-dependent internalization was required for IR-A degradation (55) (Figure 2). These findings provide a mechanistic explanation to previous studies showing that, in cells expressing only the IR-A isoform, insulin, and IGF-II induce partially different gene expression (56), downstream signaling (57), and involvement of different substrates (58).

A more recent study has identified proinsulin as a novel IR-A ligand (59). Similarly to IGF-II, proinsulin was equipotent as insulin in inducing cell proliferation in R^−^ cells expressing various levels of the IR-A, in spite of lower affinity for the IR-A and low metabolic activity (59). Degradation of both IR-A and IRS-1 was reduced after prolonged proinsulin stimulation compared to insulin action (59). If our previously described model was correct, we would expect that proinsulin and insulin would differ in their ability to promote IR-A internalization. Indeed, our preliminary data (not shown) appear to support this model by indicating that proinsulin very modestly affected IR-A internalization. Interestingly, the level of IR-A phosphorylation induced by different ligands is likely to play a more relevant role than ubiquitin in regulating receptor internalization and sorting of the IR-A for degradation (55). This is in contrast with the IGF-IR, whose ubiquitination actually enhances receptor internalization (28, 29).

Collectively, these results support the hypothesis that the lower affinity of IGF-II and proinsulin for the IR-A promotes lower IR-A phosphorylation and reduced activation of early downstream effectors compared to insulin but, at the same time, protects IR-A and IRS-1 from negative feed-back mechanisms, thereby mediating sustained and powerful mitogenic stimuli.

As we mentioned above, Grb10 binds Nedd4 (60) and promotes IGF-IR ubiquitination and internalization (28, 29). Grb10 also interacts with the IR in an insulin-dependent fashion (61–64) and Grb10 depletion by shRNA in HeLa cells inhibits insulin-dependent IR ubiquitination and degradation (65). However, whether insulin, IGF-II or proinsulin may differentially affect Grb10 and Nedd4 recruitment to the IR-A and whether Grb10 and Nedd4 may regulate IR-A internalization and sorting remains to be established. Because IGF-II and proinsulin induce lower levels of IR-A phosphorylation compared to insulin, we can speculate that the stronger mitogenic activity induced by IGF-II and proinsulin over insulin may not only be attributed to a reduced capacity to induce receptor internalization and degradation but also to a decreased ability of putative negative regulators of IR signaling, such as Grb10, Nedd4, and possibly Eps15 to bind the IR-A after IGF-II or proinsulin stimulation. Work is, therefore, ongoing in our laboratories aiming at testing this hypothesis.

However, Grb10 is not the only adaptor protein with a role in regulating IR endocytosis. Kishi et al. (66) identified APS (also called SH2B) as mediator of IR multiubiquitination in IR-overexpressing CHO cells (66). Significantly, APS-mediated IR ubiquitination enhanced IR internalization but it did not affect receptor degradation (66), suggesting a more prevalent role of APS in regulating early events of IR endocytosis than receptor sorting.

The importance of the relative affinity of the various IR ligands in regulating receptor activity has been recently confirmed by Giudice et al. (67), who showed that IGF-II stimulation of HELA cells overexpressing the IR-B induced faster receptor internalization compared to insulin. According to the model proposed, IGF-II activates proliferative responses through the endosomes, while insulin-activated IR-B would remain at the plasma membrane, where the IR-B may better interact with key molecules important for cell metabolism (67). Altogether, these data support the hypothesis that the affinity of the different ligands for the IR-A and IR-B has an important role in determining receptor fate, signaling, and downstream biological responses. However, receptor trafficking is a very complex and tightly controlled mechanism, and it is important to point out that additional mechanisms may contribute to this process, including differences of occupancy time, and stability of the different ligand–receptor complexes in the acidifying endosomal compartments, which may affect IR-A and IR-B sorting and recycling processes (68).

We have also to consider the intriguing hypothesis that the differential effect of various ligands and ubiquitin ligases on receptor internalization may serve to limit cross talk within the various receptors of the IGF-I system. However, more studies are required to further support this scenario.

## Cellular Microenvironment Regulation on IGF-IR and IR-A Action

There is increasing evidence in the literature supporting the role of the cellular microenvironment and matrix components in regulating ligand/receptor action.

The stromal-specific proteoglycan decorin has emerged in recent years as a critical regulator of tumor initiation and progression (69–71). Decorin regulates the biology of various types of cancer by modulating the activity of several tyrosine-kinase receptors involved in growth and survival. Decorin binds the EGF-R and the HGF receptor, Met, and negatively regulates their activity and signaling (72–76).

Our laboratories demonstrated that the proteoglycan decorin binds with high affinity the IGF-I, as well as IGF-IR in a region that does not overlap with the canonical binding site for IGF-I (77). Decorin exposure of urothelial cancer cells had no effect on IGF-IR phosphorylation but instead severely decreased ligand-dependent IGF-IR activation levels in a dose-dependent manner (77). In addition, prolonged exposure to decorin did not affect the stability of the IGF-IR in urothelial cancer cells either alone or in the presence of IGF-I. Significantly, decorin exposure of bladder cancer cells considerably reduced IGF-I-induced IGF-IR and caveolin-1 colocalization, suggesting that decorin may affect either IGF-IR internalization or divert the receptor into a different endocytic compartment (77). Moreover, we provided the first evidence for a role of decorin in regulating ligand-dependent stability of IRS-1 suggesting the novel hypothesis that decorin may regulate IGF-IR-dependent biological responses in bladder cancer cells not only by directly affecting receptor activation but also modulating the stability of downstream signaling proteins (77). However, the details of a possible role of decorin in modulating IGF-IR internalization have not being defined, as are the mechanisms of decorin action on IRS-1 stability.

We have more recently shown that decorin binds with high affinity both the IR-A and IR-A ligands, although the affinity for the IR-A and proinsulin was threefold lower than the affinity for IGF-II and insulin (78). Decorin did not affect ligand-mediated IR-A phosphorylation but enhanced IR-A down-regulation after prolonged IGF-II stimulation without affecting IR-A stability after insulin or proinsulin stimulation (78). In addition, decorin regulated cell surface IR-A levels by affecting insulin-dependent internalization (78). Furthermore, decorin inhibited IGF-II-mediated Akt activation without affecting insulin- and proinsulin-dependent signaling, and negatively regulated cell proliferation induced by IGF-II but not by insulin or proinsulin (78).

These results suggest that decorin effect on IR-A function substantially differs from its effect on the IGF-IR, where decorin regulates IGF-IR phosphorylation either positively or negatively in non-transformed and transformed cellular models, respectively (77, 79).

## Conclusion

The role that endocytosis plays in regulating IGF-IR and IR-A signaling has been underappreciated for several years. More recently, IGF-IR and IR endocytosis has emerged as a key step in regulating a great variety of receptor-dependent biological responses. However, much more needs to be done in order to fully appreciate how endocytosis and trafficking control IGF-IR and IR function.

The detailed knowledge of the molecular mechanisms and proteins modulating IGF-IR and IR endocytosis will greatly help in understanding how their deregulation contributes to disease.

## Conflict of Interest Statement

The authors declare that the research was conducted in the absence of any commercial or financial relationships that could be construed as a potential conflict of interest.
